# Enzyme affinity to cell types in wheat straw (*Triticum aestivum* L.) before and after hydrothermal pretreatment

**DOI:** 10.1186/1754-6834-6-54

**Published:** 2013-04-16

**Authors:** Mads AT Hansen, Budi J Hidayat, Kit K Mogensen, Martin D Jeppesen, Bodil Jørgensen, Katja S Johansen, Lisbeth G Thygesen

**Affiliations:** 1 Forest and Landscape, University of Copenhagen, Rolighedsvej 23, Frederiksberg C DK-1958, Denmark; 2 Inbicon A/S, Kraftværksvej 5, Skærbæk, Fredericia DK-7000, Denmark; 3 Department of Agriculture and Ecology/Department of Plant and Environment Sciences, University of Copenhagen, Thorvaldsensvej 40, Frederiksberg C DK-1871, Denmark; 4 Novozymes A/S, Krogshøjvej 36, Bagsværd, Denmark

**Keywords:** Cellulosic biofuels, Wheat straw, Plant tissues, Enzymatic hydrolysis, Biomass characterisation

## Abstract

**Background:**

Wheat straw used for bioethanol production varies in enzymatic digestibility according to chemical structure and composition of cell walls and tissues. In this work, the two biologically different wheat straw organs, leaves and stems, are described together with the effects of hydrothermal pretreatment on chemical composition, tissue structure, enzyme adhesion and digestion. To highlight the importance of inherent cell wall characteristics and the diverse effects of mechanical disruption and biochemical degradation, separate leaves and stems were pretreated on lab-scale and their tissue structures maintained mostly intact for image analysis. Finally, samples were enzymatically hydrolysed to correlate digestibility to chemical composition, removal of polymers, tissue composition and disruption, particle size and enzyme adhesion as a result of pretreatment and wax removal. For comparison, industrially pretreated wheat straw from Inbicon A/S was included in all the experiments.

**Results:**

Within the same range of pretreatment severities, industrial pretreatment resulted in most hemicellulose and epicuticular wax/cutin removal compared to lab-scale pretreated leaves and stems but also in most re-deposition of lignin on the surface. Tissues were furthermore degraded from tissues into individual cells while lab-scale pretreated samples were structurally almost intact. In both raw leaves and stems, endoglucanase and exoglucanase adhered most to parenchyma cells; after pretreatment, to epidermal cells in all the samples. Despite heavy tissue disruption, industrially pretreated samples were not as susceptible to enzymatic digestion as lab-scale pretreated leaves while lab-scale pretreated stems were the least digestible.

**Conclusions:**

Despite preferential enzyme adhesion to epidermal cells after hydrothermal pretreatment, our results suggest that the single most important factor determining wheat straw digestibility is the fraction of parenchyma cells rather than effective tissue disruption.

## Background

Increasingly expensive fossil fuels and environmentally perilous greenhouse gas emissions have initiated research in alternative and more sustainable energy sources. One alternative is hydrothermal and enzymatic degradation of wheat straw (*Triticum aestivum* L.) into fermentable sugars and subsequent conversion into bioethanol, ready for use in the transport sector.

Four main processes are involved in biochemical conversion of wheat straw into bioethanol: pretreatment, saccharification, fermentation and distillation [1,2]. Each stage has separate process parameters: 1) biological; choice of biomass and enzyme mix, 2) chemical; degree of polymerisation (DP), crystallinity and exposure of mainly cellulose, hemicellulose and lignin, 3) physico-chemical; solubilisation and transformation of polymers and 4) physical; moisture content, particle and pore size and specific surface area [3-6]. In theory, all of these parameters may affect the digestibility of various organs and tissues differently within the same plant species.

**Figure 1:**
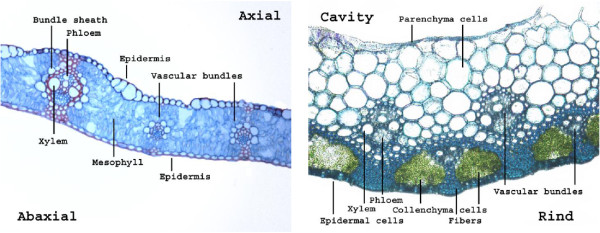
**Wheat straw anatomy.** LM images of wheat leaves and stems. Note the high content of mesophyll in leaves (**left**), consisting of parenchyma cells, and less dense tissues compared to stems (**right**).

The two main organs in wheat straw are leaves and stems both of which volume-wise primarily consist of parenchyma cells (see Figure 1). Parenchyma cells often have only primary walls and no lignified secondary wall thickenings [7]. In stems, parenchyma cells constitute the cortex and account for roughly half the tissue volume; in leaves, they constitute the mesophyll and vascular bundle sheaths [8]. In wheat, vascular bundles consist of xylem (tracheids) and phloem (sieve tube elements) and are supported by sclerenchyma tissue (fibers) in stems. Tracheids and fibers both have thickened and often lignified secondary walls. Dermal tissues (epidermis) envelop both leaves and stems and are covered by a cuticle consisting of cutin overlaid by wax deposits [7-12]. Inside the stem is the intermodal cavity (lacuna), sheathed by a thin modified layer of parenchyma cells called the pith cavity lining (PCL) [13]. Fibers, dermal tissues and silicates together provide rigidity and protection from degradation by weather conditions, water loss, microbes and insects etc. [14,15].

Investigations show that grass tissues vary significantly in digestibility [2,16-18]. Incubated with rumen cultures, parenchyma cells were the most digestible followed by phloem [1]. This was also observed in pretreated sections of wheat stems using commercially available enzymes [19]. Cellulose microfibrils are presumably more directly accessible to enzymatic attack in primary cell walls with little or no lignification. It can thus be speculated that a diminishing fraction of parenchyma cells is a contributing factor [19] to the steady decrease in glucan conversion rate as enzymatic hydrolysis progresses over time [20].

Disruption of tissue structures, decrease in polymers’ DP and increase in the available surface area is supposed to enhance enzymatic digestibility [21] although the significance of particle size reduction is disputed [3,5]. In high solids processes though, effective liquefaction is necessary and particle size reduction a prerequisite [22,23].

For lignin containing tissues, unproductive binding of enzymes to surface lignin is considered a limiting factor during hydrolysis although significant amounts of cellulolytic enzymes desorb when fresh substrate is added [24,25]. Particularly carbohydrate-binding modules (CBMs), found in many cellulolytic enzymes, attaching the enzyme to the lignocellulose surface [26] via hydrophobic interactions [27], have been suspected of contributing to unproductive binding to lignin. Yet studies of cellulases from *Trichoderma reesei* have shown that endoglucanase (EG) II (Cel5A, family GH5), which has a more open catalytic domain (CD), adsorbed more readily to lignin than cellobiohydrolase (CBH) I (Cel7A, family GH7) even though the former had had its CBM removed [28,29]. Enzyme adsorption is complex and affected not only by the enzyme’s mode of action and tertiary structure [30] but also by the substrate’s chemical and physical dynamics during the degradation processes [31,32].

**Figure 2:**
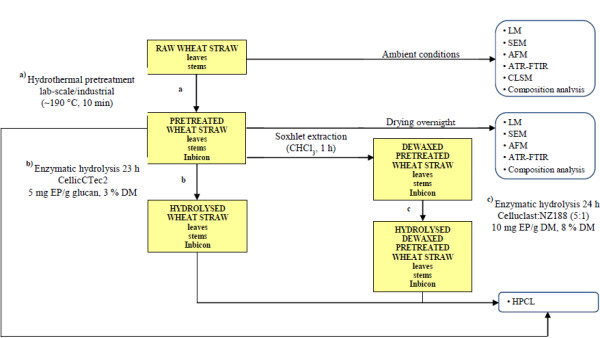
Experimental setup.

In this paper, the two biologically different wheat organs, leaves and stems, were separated and subjected to lab-scale hydrothermal pretreatment in a customised reactor [19]. This allowed for the structural architecture of the tissues to be preserved and individually studied by image analysis. Afterwards, one set of pretreated samples was further dewaxed to investigate the effects of cutin and epicuticular waxes and on enzymatic hydrolysis. All samples were then enzymatically hydrolysed to compare their relative digestibility according to a number of parameters, including chemical composition and removal of lignin, hemicellulose, cutin and epicuticular waxes, tissue composition and structural disruption, reduction in particle size and changes in enzyme adhesion. For the study of enzyme adhesion, cross sections of leaves and stems and samples of the three principal and distinctive cell types in wheat straw (fiber cells, epidermal cells and parenchyma cells) before and after pretreatment were incubated with a fluorescently labelled monocomponent endoglucanase and exoglucanase. For comparison, industrially pretreated wheat straw from Inbicon A/S (http://www.inbicon.com) was included in all the experiments. The experimental setup is schematically presented in Figure 2.

The aim was to study the effects of hydrothermal pretreatment on enzyme adhesion and digestibility. This was done in order to highlight the relative importance of inherent cell wall characteristics and the diverse effects of mechanical disruption and changes in chemical composition.

## Results and discussion

### Chemical composition

In wheat, vascular and sclerenchyma tissues are most abundant in stems. These two tissues have more secondary wall thickenings where xylan plays a crucial role [33] together with lignin [34]. This is reflected in the composition analysis in Table 1 where raw stems had more lignin and xylan compared to leaves. After pretreatment, leaves had more lignin than stems. According to some studies, increasing amounts of lignin at increasing pretreatment severities is due to ‘pseudo-lignin’ which consists of condensation products of lignin and other compounds such as proteins, furfural and other extractives but is detected as lignin in the composition analysis. This phenomenon has been reported in fractionated poplar (*Populus tremoloides* Michx.) [35] and wheat straw [36] where the amounts of lignin were significantly increased after treatment. According to our composition analysis however, lignin contents in the solid fractions will arithmatically increase in any case as hemicellulose, salts, waxes, cutin etc. are removed during pretreatment.

**Table 1:** Chemical composition of raw and pretreated wheat leaves and stems

	**Glucan**	**Xylan**	**Arabinan**	**Galactan**	**Lignin**	**Ash**	**Mass balance**
**Raw**							
Leaves	40.0 ± 0.8	14.3 ± 1.0	2.9 ± 1.0	1.3 ± 0.2	18.4 ± 1.0	7.4 ± 0.0	84.2
Stems	42.3 ± 0.1	20.1 ± 0.7	3.0 ± 0.0	0.9 ± 0.0	22.4 ± 2.5	5.1 ± 0.1	93.8
**Pretreated**							
Leaves	57.2 ± 1.9	11.7 ± 0.3	0.5 ± 0.0	0.3 ± 0.0	24.7 ± 0.7	4.7 ± 0.8	100.5
Stems	59.1 ± 1.3	14.4 ± 0.8	0.5 ± 0.1	0.1 ± 0.0	21.1 ± 1.2	1.2 ± 0.2	96.4
Inbicon^a^	^*^56.4 ± 0.7	^*^6.5 ± 0.5	n.d.	n.d.	29.6 ± 0.5	2.9 ± 0.2	95.3

^a^Severity factor = 4.02.

^*^Glucan/xylan content for Inbicon pretreated wheat straw at SF = 3.54 and 3.72 were 49.3/12.3 and 51.7/7.7, respectively.

Values are averages in % ± stdev. (n = 3).

**Figure 3:**
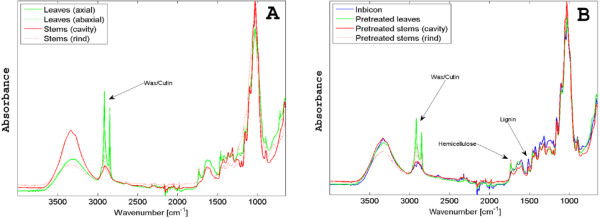
**IR spectra.** ATR-FTIR spectra of raw (**A**) and lab-scale pretreated (**B**) wheat leaves and stems and Inbicon pretreated wheat straw.

Leaves contained slightly more ash and significantly more residuals than stems. According to literature, minerals in wheat straw are primarily silica (SiO_2_) [14] while cutin and epicuticular waxes, together with proteins, pectins and lipids etc., account for most of the residuals [37]. None of the residuals were individually quantified in this study, though. Cutin and epicuticular waxes consist of hydroxylated and esterified aliphatic acids and long-chain fatty acids, respectively [10,37,38]. Therefore, the strong infrared (IR) absorption bands for leaves especially at ca. 2915 and 2850 cm^-1^ in Figure 3, representing aliphatic CH_2_ groups [38], most likely originate from cutin and wax. Our IR analysis confirms earlier results, that the industrial pretreatment successfully removes surface wax [39] while lab-scale pretreatment is much less efficient.

After pretreatment, stems contained more hemicellulose compared to leaves (Table 1). This was expected as stems have more secondary walled tissues with relatively large ratios of hemicellulose [33,34], which together with none the least lignin helps protect the cellulose [5,9]. Leaves had relatively more lignin, virtually no residuals and less hemicellulose and ash compared to stems. Lack of residuals in leaves (such as aliphatic compounds) is in poor accordance with the IR analysis but could be due to strong surface adhesion of those residuals not solubilised and washed away after pretreatment (see below).

Together with Table 1, the IR spectra confirm that industrial pretreatment effectively removes hemicellulose but also increases the amount of surface lignin as witnessed by the aromatic skeletal vibrations around 1510 cm^-1^ and 1600 cm^-1^[40]. According to previous investigations, lignin coverage partly shields the cellulose from enzymatic attack by physically blocking access [19,41,42]. On the other hand, extraction of lignin also results in increased access to otherwise sealed cellulose within the cell wall matrix, thus increasing digestibility [43-45].

### Structural integrity

**Figure 4:**
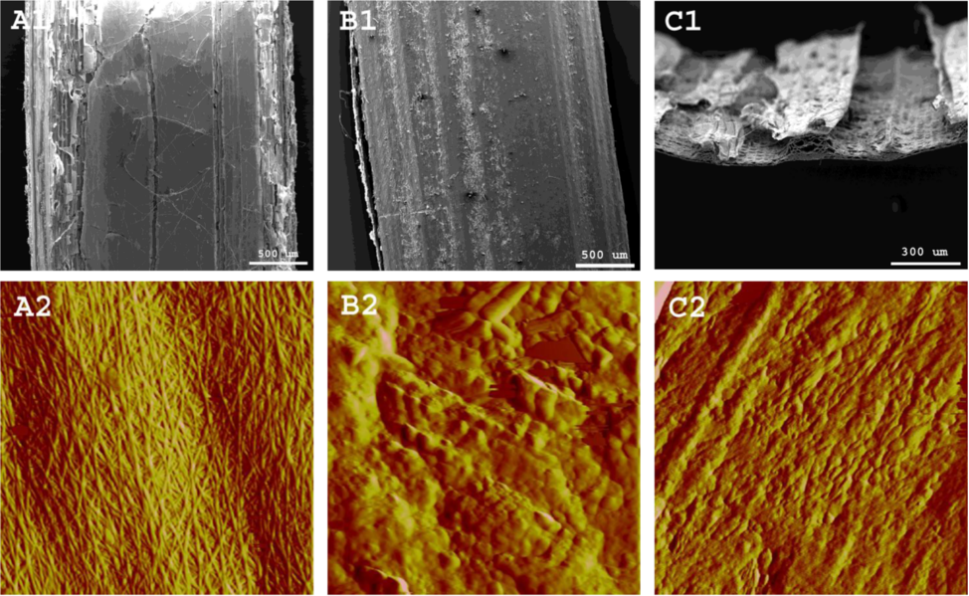
**SEM and AFM.** SEM (**1**) and 2 μm^2^ AFM (**2**) images of raw wheat stems (**A**, cavity; **B**, rind) and leaves (**C**). On the AFM images, note the surface of microfibrils on the stem cavity and the lumpy ridges interpreted as cutin and epicuticular waxes partly on the stem rind and on the leaves.

On the scanning electron microscope (SEM) images of raw stems and leaves (Figure 4), the stem cavity and leaf appear brittle compared to the stem rind. On the atomic force microscope (AFM) images, the stem rind and leaf adaxial and abaxial side (indistinguishable, not shown)both had ridges of cutin and wax, c.f. with Figure 3A. We still believe that leaves, together with the stem cavities and cortices, must be significantly less recalcitrant than the stem rind as the cell walls in these tissues mainly consist of relatively accessible cellulose microfibrils (see above).

**Figure 5:**
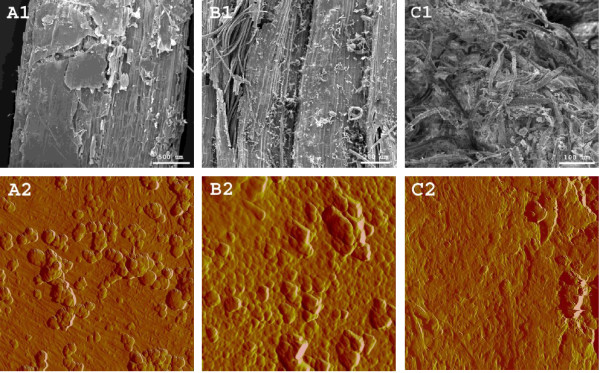
**SEM and AFM.** SEM (**1**) and 2 μm^2^ AFM (**2**) images of lab-scale pretreated stem (**A**) and leaves (**B**) and Inbicon (**C**) pretreated wheat straw. On the AFM images, microfibrils in the stem cavity are significantly covered in what is interpreted as lignin globules.

According to Figure 5, lab-scale pretreated leaves were more severely disrupted and had more visible debris compared to lab-scale pretreated stems (B1 and A1, respectively); AFM showed no distinct cellulosic features but rather a remaining layer of aliphatic wax/cutin, cf. with Figure 3B and Figure 4B2. The stems’ cavity lining was also disrupted together with much of the exposed underlying cortex while the rind was structurally unaffected (rind not shown). As represented in Figure 5A2, stem cavities also had significant amounts of droplets on the cell wall surface which in previous studies have been interpreted as extracted and re-deposited lignin [39,41,45] while in Figure 5B2, the dominating feature on the leaves is interpreted as morphologically modified cutin and waxes (see Figure 3B). The tissue structures of whole wheat straw pretreated at Inbicon was completely disrupted (see Figure 5C1), exposing all cell types at random for enzymatic degradation. Contrary to the lab-scale samples that were kept static in blue cap bottles during pretreatment, industrial pretreatment involved mechanical mixing via a sluice system and screw press. Thus, industrially pretreated wheat straw was thoroughly disintegrated and the particle sizes virtually reduced to individual cells. The IR spectra i Figure 3 showed more lignin on the surface although this was not distinctly visible on the AFM images in Figure 5C2.

Changes in cellulose crystallinity after pretreatment as a result of aggregation of cellulose fibrils and hemicellulose removal have been reported (see above). Visually, no clear change in cellulose fibril aggregation was observed, only deposition of lignin on the surface (Figure 5A2). Correlations between cell type and crystallinity in wheat straw specifically, have to the best of the authors’ knowledge not been sufficiently elucidated. When addressed, the unspecific and general term ‘fiber’ is often used for any plant cell. Two studies though, have investigated the crystallinity of raw Moso Bamboo (*Phyllostachys adulis* (Carr.) H. De Lehaie) and acid and alkaline sulphite pretreated Royal palm sheaths in relation to fiber and parenchyma cells [46,47]. In the former, crystallinity increased as the fiber fraction increased from the inner to the outer part and the parenchyma fraction decreased; in the latter, parenchyma cells had lower crystallinity indices than fibers as well as less lignin and hemicellulose. The studies concluded that their biomass material was anatomically differentiated and unevenly digestible. A third study investigated hydrothermally pretreated wheat straw under more or less the same conditions as this study and found no adverse effects on cellulose crystallinity [39]. However, the effect of cellulose crystallinity on enzymatic hydrolysis is disputed and some studies argue that the overall yield correlate significantly better with particle size [48].

A possible side effect of pretreatment is hornification. Whether partial lignin extraction and hemicellulose removal during pretreatment of wheat straw during hydrothermal pretreatment [39,41,45] followed by pressing to increase dry matter (DM) content [49] has a negative effect on enzymatic digestibility [50-52] could be speculated but are not quantified in this study. Pressing-induced hornification could reduce enzyme digestibility [53] but is negligible when solids content is only increased to ca. 40% [53], well above the DM content in our case (see below).

### Enzyme adhesion

Enzyme-substrate interactions are determined not only by the chemical composition of the cell wall but to a large extend also by the specific architecture of the cell wall matrix [54]; within a given plant species, cell wall architecture varies between cell types and tissues and plant maturity. It is therefore likely that different cell types are differently affected during pretreatment and that the affinity of cellulolytic enzymes to the cell wall surfaces also changes. To study this, cross sections of raw leaves and stems as well as pretreated samples were incubated with a monocomponent fluorescently labelled exoglucanase or endoglucanases and imaged by confocal laser scanning microscope (CLSM). The two enzymes have the same type of CBM (CBM1) but different CDs; the exoglucanase (family GH7 from *Hypocrea jecorina*) has a tunnel-shaped active site region within the CD [55], though still capable of some endo-acting activity [56,57], while the endoglucanase (family GH45 from *Humicola insolens*) has an active site within an open cleft of the CD [58].

**Figure 6:**
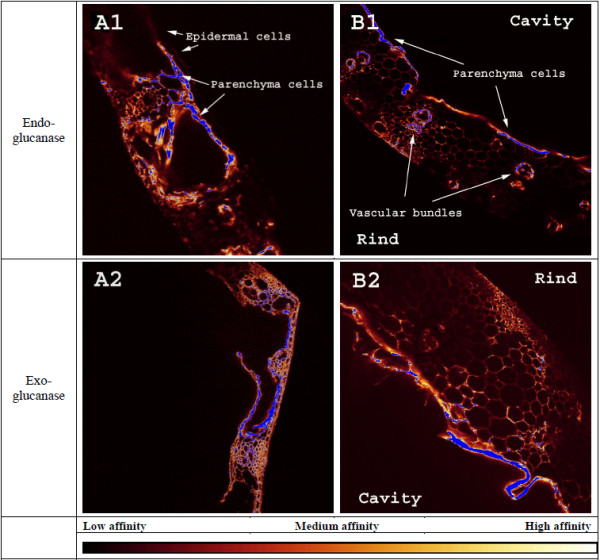
**Enzyme affinity towards raw samples.** CLSM images, roughly 1 mm^2^, of raw leaves (**A**) and stems (**B**) incubated with a fluorescently labelled endoglucanase (**1**) and exoglucanase (**2**). Enzyme adhesion is not quantitatively comparable, only relative. Blue colour indicates off-scale high adhesion. Note the relatively high affinity to parenchyma cells and low affinity to epidermal cells that cover the stem rind and both sides of the leaf.

In Figure 6 it should be stressed that the raw samples had been sectioned and the cell walls’ cross-sections thereby exposed to enzymatic adhesion, a different condition compared to the cells in the pretreated samples where only the outer cell walls were exposed. This was because, as a result of the pretreatment, these samples had become too soft for sectioning which would otherwise severely disrupt them before the glass slide mounting. Additionally, the incubation temperature for both raw and pretreated samples was 4°C, another fundamentally different condition than industrial *in situ* enzymatic hydrolysis where the temperature is significantly higher (see below). In this study however, we assumed the enzyme adhesion was not significantly influenced by the sectioning and incubation temperature and that the raw and pretreated samples were comparable.

**Figure 7:**
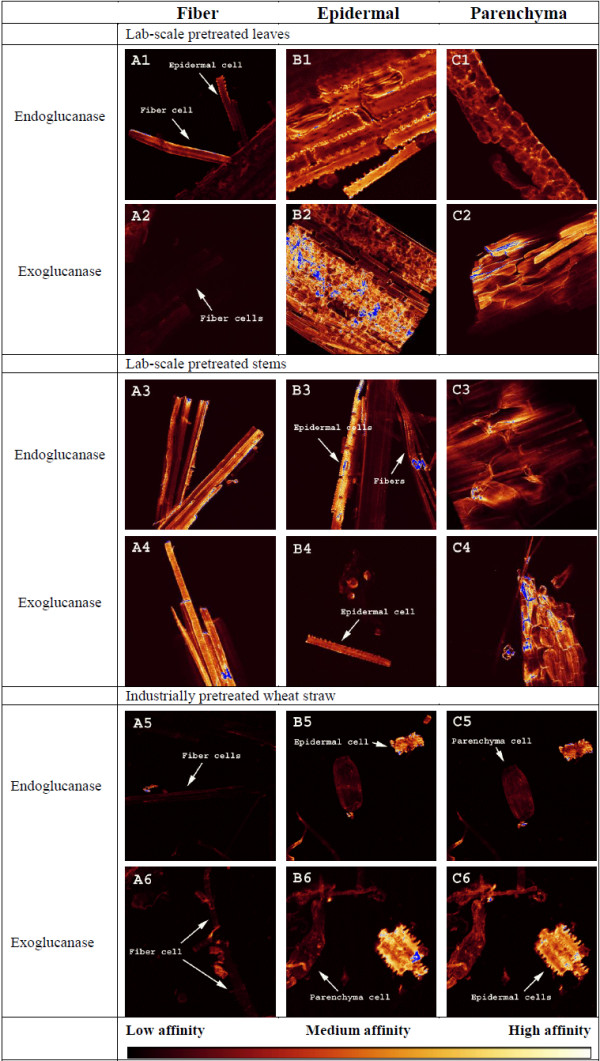
**Enzyme affinity towards pretreated samples.** CLSM images of lab-scale pretreated wheat leaves (**1, 2**), stems (**3, 4**) and industrially pretreated wheat straw (**5, 6**) incubated with a fluorescently labelled endoglucanase (**1, 3, 5**) or exoglucanase (**2, 4, 6**) showing the level of adhesion to fiber (**A**), epidermal (**B**) and parenchyma (**C**) cells. In order to facilitate comparison, all images were taken with the same objective (10x) and setting of laser intensity (20%) and gain (500 V). Blue indicates off-scale high adhesion. Images were taken at the level where the emission intensity was highest for the cell type of interest. Note the high affinity to epidermal cells compared to fibers and parenchyma cells after pretreatment.

Images of the raw cross sections in Figure 6 show that endoglucanase barely adheres to epidermal cells in any of the two organs while both enzymes adhere most to parenchyma cells. Similarly, a study of dried corn stover stems found that tissues containing plant cells with only primary walls were highly accessible to cellulases while tissues with secondary walls were not [59]. However, after pretreatment both enzymes adhered extensively more to epidermal cells, especially in the industrially pretreated samples (see Figure 7). Epicuticular waxes have relatively low melting points, well below those applied in our experiments [60,61], and an earlier study showed that industrial hydrothermal pretreatment partly removes waxes on wheat straw [39] and Figure 3 shows a decrease in cutin/wax after lab-scale pretreatment also. So, some degree of solubilisation, chemical modification or relocation must have taken place to cause this shift in enzyme adhesion. But, to the best of the authors’ knowledge, the exact effect of waxes on enzymatic hydrolysis has not yet been investigated. Although, according to studies on spruce and poplar foliage, tissue damage increases as epicuticular waxes are removed [62-64]. Consequently, wax removal should in theory increase the sugar yield although this was not the case according to our results (see below).

**Figure 8:**
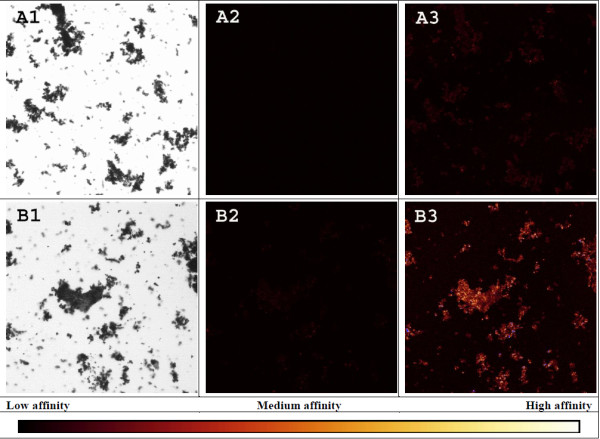
**Enzyme affinity towards purified lignin.** LM (**1**) and CLSM images (**2, 3**) of purified lignin incubated with a fluorescently labelled endoglucanase (**A**) or exoglucanase (**B**). Each row shows one identical motive taken with the same objective (4x). CLSM images were captured using the same laser intensity of 20% but with different gains, i.e. 500 V (**2**) and 700 V (**3**), respectively. Notice that no emission is detected at ‘standard’ 500 V, only after a significant increase in gain to 700 V.

Hydrothermal pretreatment changes cell wall properties so that cellulases used in this experiment adhere significantly more to epidermal cells. Figure 5A2 suggests that the accessible surface area of parenchyma cells might have been significantly reduced by the presence of droplets, interpreted as extracted and re-deposited lignin, which hinder enzyme access and adhesion to the cell wall. To visualise the level of enzyme binding to lignin, enzymatically purified lignin, i.e. cellulolytic lignin (CL), was incubated with the same fluorescently labelled enzymes. As seen in Figure 8, the emission signal needed significant enhancement for any adhesion to be detected, indicating that enzyme binding to lignin only is negligible compared to tissues in general. Other studies also conclude that cellulase adhesion to lignin is negligible [59] and that it has minimal effects on overall conversion yields [65,66]. Cell type specific enzyme adhesion during wheat straw degradation has to our knowledge barely been addressed and the exact cause can only be speculated.

### Sugar yield

**Figure 9:**
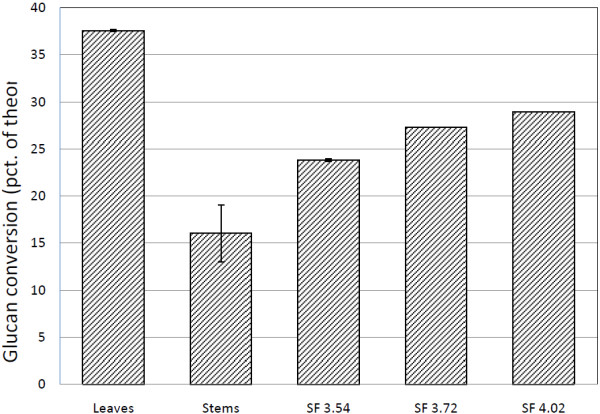
**Glucan conversions of pretreated samples.** Conversion of % theoretical glucan in lab-scale pretreated wheat leaves and stems (average SF = 3.93) and Inbicon pretreated wheat straw at various severities. Samples were hydrolysed for 23 h, using 5 mg EP Cellic CTec2 pr. g glucan.

Lab-scale pretreated leaves had the highest glucan conversion after 23 h of enzymatic hydrolysis using Cellic CTec2, equal to approximately 38% of the theoretical content (see Figure 9). Next to leaves, industrially pretreated wheat straw had 24–29% glucan conversion depending on severity factor (SF) [67]. Lab-scale pretreated stems only had 16% on average. The average SF for both lab-scale and industrially pretreated samples was within the same range, i.e. 3.93 and 3.54-4.02 respectively. The low digestibility of stems correlates with the relatively intact tissues after lab-scale pretreatment [19] which hinders enzyme access during hydrolysis [5]. Also, when comparing raw and pretreated samples in Table 1 and Figure 3, lab-scale pretreatment caused less chemical and physical changes than industrial pretreatment. Despite that, industrially pretreated samples were still less digestible than lab-scale pretreated leaves even when treated at slightly higher severities. Whole wheat straw consists of more than 80% w/w stem [68] and when baled, some of the dry and volatile leaf material is lost. This implies that industrially pretreated samples tissue-wise mostly consist of stems. Compared to other investigations of enzymatic conversion of wheat straw [22,36] and corn stover [69], our conversions are very low. This was expected though, as neither enzyme dosage, DM content nor residence time was optimised in this study.

**Figure 10:**
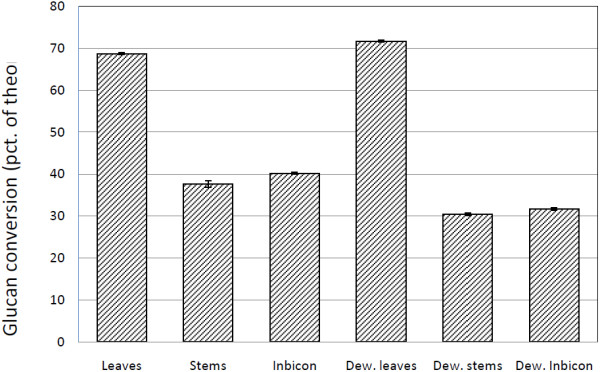
**Glucan conversions of dewaxed pretreated samples.** Conversion of % theoretical glucan in lab-scale pretreated wheat leaves and stems and Inbicon pretreated wheat straw after 24 h using 10 mg Celluclast:NZ188 (5:1) pr. g DM. Dew. = dewaxed pretreated samples.

As mentioned earlier, epidermal tissues in wheat straw are covered by a protective layer of cutin and waxes. Yet when dewaxed by chloroform and air-dried at 65°C, no significant differences in glucan conversions could be seen (data not shown). To rule out the possibility of drying-induced hornification, another set of samples were washed in ethanol and subsequently water (see below) immediately after dewaxing. But, these samples also showed either negligible or outright negative effects on the sugar yield (see Figure 10).

The most striking effect of industrial scale hydrothermal pretreatment is the tissue disruption and reduction of particle size from tissues into individual cells, most likely one of the reasons why industrially pretreated samples are more digestible than lab-scale pretreated stems. However, significant chemical differences were also found by compositional and IR analyses, showing hemicellulose removal to be much more effective during industrial pretreatment. So whether structural integrity or chemical composition affect sugar yield the most still require further investigations. Our results showed increased enzyme adhesion to epidermal cells after hydrothermal pretreatment but no increase in conversion yields upon dewaxing; perhaps the fraction of epidermal cells is too small to influence the overall sugar yield. More investigations are needed to clarify this issue.

## Conclusions

Lab-scale pretreated wheat leaves had significant amounts of surface aliphatic compounds stemming from cutin and waxes while whole wheat straw, industrially pretreated at Inbicon, had most surface lignin and contained least hemicellulose. The removal and extraction of hemicellulose and lignin, respectively, during pretreatment was also most evident in the industrially pretreated samples as were structural breakdown of tissues which might have increased cellulose crystallinity. Despite being structurally intact prior to enzymatic hydrolysis, lab-scale pretreated leaves had the highest glucan conversion followed by industrially pretreated wheat straw. Lab-scale pretreated stems had the lowest glucan conversion and were also structurally the most intact sample. The observed increased adhesion of exoglucanase and endoglucanase to epidermal cells after hydrothermal pretreatment were quantitatively insignificant as wax removal had no positive effects on enzymatic conversion. Hence, further investigations are needed to elucidate whether the increased adhesion is productive or not. According to our findings, the single most significant factor determining digestibility seems to be tissue composition as lab-scale pretreated leaves had the largest share of parenchyma cells.

## Methods

Separated wheat leaves and stems were hydrothermally pretreated on lab-scale and structurally and chemically analysed together with industrially pretreated wheat straw from Inbicon. Samples were then enzymatically hydrolysed, as were dewaxed pretreated samples, and the solubilised sugars quantified. Finally, the adhesion of two fluorescently labelled monocomponent cellulases to well-defined tissues and cell types from all pretreated samples was investigated.

### Wheat straw

Wheat straw for lab-scale pretreatment was collected at Tystofte (Denmark) in 2006 and stored at ambient conditions with a DM content of ca. 90% (% w/w). Wheat straw for industrial pretreatment at Inbicon was grown in Denmark, dried in the field and stored as bales at ambient conditions. Composition analyses of raw and pretreated leaves without sheaths, stems and industrially pretreated whole wheat straw were performed in triplicate according to the procedure described by The National Renewable Energy Laboratory (NREL) [70].

### Pretreatment

For the lab-scale pretreatment, separated raw leaves and stems were sectioned into ca. 5 cm pieces and 3 g placed in blue cap bottles together with 80 mL demineralised water. Each bottle was then placed in a custom made high pressure reactor and heated in an oil bath up to 185°C. The heat-up time was ca. 50 min. Samples were further pretreated for 10 min in which the temperature inside the chamber would rise to ca. 190–195°C. No mechanical disruption of the samples took place during the treatment. Upon cooling, samples were stored together with the liquid.

The industrially pretreated samples of whole wheat straw were provided by the Inbicon pilot plant in Skærbæk, Denmark. There, the raw material had been cut into ca. 5 cm long pieces and soaked in liquid from the later pretreatment reactor to a DM content of 20–40%. It was then fed to the pretreatment reactor at a rate of 50 kg pr. h and mechanically moved via a patented sluice system. The hydrothermal pretreatment was performed at a water-to-straw ratio of 5:1 with a residence time of 12 min and temperature at 180–195°C by injection of steam. Afterwards, the pretreated material was delivered to a screw press and washed with ca. 70°C hot water and separated in another screw press into a solid fraction with higher DM content, containing more than 90% of the cellulose and the majority of the lignin, and a liquid fraction, containing the majority of salts and solubilised hemicellulose [71].

To compare the severity of lab- and industrial scale pretreatments, the semi-empirical parameter, the severity factor (SF = log(R_0_)), was used. SF is comprised of the reaction time (*t*) in min, the reaction temperature (*T*) in °C and the reference temperature (*T*_*ref*_,) in °C, in this case 100°C [67].

$$\mathrm{SF}=\log_{10}\left[R_{0}\right]=\log_{10}\left[t.e^{\frac{T-T_{\text{ref}}}{14.75}}\right]$$

The heat-up time for the lab-scale experiments was included by summarizing in five min intervals the time of reaction after the temperature exceeded 100°C:

$$\log_{10}\left[R_{0}\right]=\log_{10}\left[\overset{t_{T>100\;\mathrm{{}^{\circ}C}}}{\underset{t_{\text{end}}}{\sum }}R_{0}\left(t\right).\Delta t_{5\;\text{min}}\right]$$

### Dewaxing

A subset of separated lab-scale pretreated leaves, stems and Inbicon pretreated wheat straw was washed three times in demineralised water prior to Soxhlet extraction for 1 h using 350 mL of chloroform as solvent per ca. 10 g sample. Upon cooling, all samples were washed three times in ethanol, six times in demineralised water and kept moist until enzymatic hydrolysis.

### Enzymatic hydrolysis

Enzymatic hydrolysis of lab-scale pretreated leaves and stems and Inbicon pretreated samples was performed in triplicates, conducted in 50 ml Nunc tubes (http://www.thermoscientific.com) at 50°C using Cellic CTec2 (Novozymes A/S, Bagsværd, Denmark). The density and specific activity of the enzyme mix was 1.18 g/mL and 120 FPU/g, respectively and the enzyme dosage adjusted to 5 mg enzyme protein (EP) pr. g glucan. DM was adjusted by 0.1 M sodium citrate buffer solution to the lowest percentage of all the samples, i.e. 3% equal to that of leaves immediately after pretreatment and pH adjusted to 4.7-5.3 by sodium hydroxide. During hydrolysis, the tubes were shaken at 205 rpm.

Enzymatic hydrolysis of the dewaxed samples was performed in triplicates and the DM content adjusted to the lowest percentage of all the samples, i.e. 8% equal to that of leaves, using 50 mM sodium-citrate buffer, pH 4.8. Enzymes were Celluclast 1.5 L and Novozyme 188 (Novozymes) with a weight ratio of 5:1 and protein content of 130 (specific activity 72 FPU/mL) and 220 mg/mL, respectively, as measured by the ninhydrin protein assay using BSA as reference [72]. The enzyme mix was then diluted, resulting in 10 mg protein/g DM (specific activity equal to 5 FPU/mL). 0.5 mL was added to triplicates of 0.5 g DM from each sample, placed in 20 mL plastic flasks. Samples were then hydrolysed for 24 h at 50°C while tumbled [22].

The content of mono- and disaccharides after hydrolysis of all the samples (d-glucose, d-xylose, l-arabinose and d-cellobiose) was quantified using an Ultimate HPLC system (Dionex Corporation, Sunnyvale, CA) equipped with a Shimadzu RI-detector (Shimadzu Corporation, Kyoto, Japan). The separation was performed in a Phenomenex Rezex ROA column (Phenomenex Inc., Torrance, CA) at 80ºC with 5 mM H_2_SO_4_ as eluent at a flow rate of 0.6 mL/min. Samples were filtered through a 0.22 or 0.45 μm filter and diluted with eluent before analysis. The average content of cellobiose and glucose in all samples, including that for the composition analyses, was summarised as cellulose and the average content of xylose and arabinose as hemicellulose. Hydration of glucose and cellobiose to cellulose was corrected for by a factor of 0.9 and 0.95, respectively and for xylose to hemicellulose by a factor of 0.88.

### ATR-FTIR

Attenuated total reflectance-Fourier transform infrared (ATR-FTIR) spectra were obtained in triplicates using a ThermoFischer Scientific Nicolet 6700 FTIR spectrometer (ThermoFischer Scientific, Waltham, MA) equipped with a Golden gate ATR accessory (http://www.specac.com). Spectra from 4000 to 600 cm^-1^ were obtained with a 4 cm^-1^ resolution, 200 background scans and 100 scans for each sample spectrum. Each spectrum was background corrected using the standard normal variate (SNV) [73]. Lastly, the average of the three SNV corrected spectra was calculated for each type of sample.

### Microscopy

For microscopy analyses, raw wheat straw samples were imaged as they were upon storage at ambient conditions; lab-scale pretreated samples were washed in demineralised water and freeze dried directly afterwards while the Inbicon pretreated samples were freeze dried as they were.

Prior to SEM imaging, all samples were coated with gold/palladium (Au/Pd) by a SC7640 Suto-/Manual High Resolution Sputter Coater (Quorum Technologies, Newhaven, UK) before imaged with a FEI Quanta 200 (FEI Company, Eindhoven, The Netherlands) SEM, operated at 10–20 kV.

For AFM imaging, a MultiMode scanning probe microscope with a Nanoscope IIIa controller (Veeco Instruments Inc, Santa Barbara, CA) was used operating in TappingMode with an etched silicon probe (MPP-12100, Veeco NanoProbe, Santa Barbara, CA). The resonance frequency was 150–300 kHz and the scan rate 0.5-1.97 Hz. The drive amplitude and amplitude setpoint were continually adjusted during scanning to avoid artefacts.

For light microscopy images, 80 μm sections of wheat stems were made on a Leica VT 1000S vibratome (Leica Microsystems A/S, All Microscopy and Histopathology Instrumentation, Ballerup, Denmark) and embedded in 5% agarose in phosphate buffer, pH 7. Sections were then stained with Toluidin Blue [74] for 5 min and washed in water 3 times before viewing in a Leica DMR fluorescence microscope and fitted with a Leica DC 300 F camera (both Leica Microsystems A/S, All Microscopy and Histopathology Instrumentation, Ballerup, Denmark). The wheat leaf was from an anatomical collection of plant tissues for teaching, imaged using the same Leica microscope.

The Confocal Laser Scanning Microscope (CLSM) was a Leica TCS SP2 (http://www.leica-microsystems.com), equipped with a 10x objective, 0.4 NA throughout all the experiments. Excitation was performed using a 633 nm laser source at 20% power, generally with 500 V gain. Emissions were collected between 650–750 nm. For clarity, some of the images were electronically magnified. This did not, however, alter the intensity of the signal significantly (data not shown). Images were colour coded according to the intensity of light emission from black (none) over brown-red (mediocre) to yellow and white (maximum) while blue signified signal overload. An exoglucanase (family GH7 from *Hypocrea jecorina*) and an endoglucanase (family GH45 from *Humicola insolens*) were provided by Novozymes, Bagsværd, Denmark in chromatographically pure form. The enzymes were incubated overnight with fluorophore Dylight 633™ (Thermo Scientific, Rockford, IL) in 0.05 M borate buffer, pH 8.5 at 4°C. Excess fluorophore was removed via multiple centrifugations in Vivaspin ultrafiltration spin columns (5 K MWCO, Sartorius Stedim GmbH, Göttingen, Germany) at 4,700 rpm for 15 min until a clear supernatant was obtained. At the same time the buffer was exchanged with MQ water. A ca. 10^-5^ M stock solution of labelled enzymes in MQ water was obtained with a fluorophore to protein mole ratio of 1:1 (exoglucanase) and 1.4:1 (endoglucanase). Prior to use, stock solutions of the exoglucanase and endoglucanase were diluted 14 and 20 times, respectively in a 0.1 M Na-acetate buffer, pH 4.8.

For transversal microscopic analysis only raw leaf and stem could be successfully mounted; lab-scale and industrially pretreated samples were too fragile. Slices ca. 0.3 mm thick were incubated in labelled enzyme solution overnight at 4°C and briefly washed with MQ water prior to mounting on glass slides. Glass slides with a depression in the middle were used to facilitate mounting of thick specimens. For longitudinal microscopic analysis, both raw and pretreated samples were dissected and incubated in enzyme solution overnight at 4°C. Following a brief washing with MQ water, selected tissues were mounted.

The CL derived from processed wheat straw from the Inbicon plant, further purified by enzymatic hydrolysis by 75 FPU/g DM Celluclast:NZ188 (5:1) (Novozymes A/S) in 50 mM sodium-citrate buffer while stirred in an incubator at 50°C for 72 h. The precipitate was then separated and cleansed by three repetitions of centrifugation and washing in MQ water. The precipitate was afterwards added 3 μL alcalase (Novozymes A/S) per mL of 100 mM sodium-citrate buffer, pH 7 at 5% DM, stirred in an incubator at 50°C for 20 h and cleansed by three repetitions of centrifugation and washing in MQ water. Both steps (adding cellulases followed by alcalases) were performed three times. The microscopic analysis was then performed as described above.

## Abbreviations

AFM: Atomic force microscopy; ATR-FTIR: Attenuated Total Reflectance-Fourier Transform Infrared; CBH: Cellobiohydrolase; CBM: Cellulose binding module; CD: Catalytic domain; CL: Cellulolytic lignin; CLSM: Confocal laser scanning microscopy; DM: Dry matter; DP: Degree of polymerisation; EG: Endoglucanase; EP: Enzyme protein; LM: Light microscopy; SEM: Scanning electron microscopy; SF: Severity factor.

## Competing interests

Co-author KSJ is an employee at Novozymes A/S and supplied the monocomponent enzymes for the CLSM studies; KKM and MDJ are employees at DONG Energy A/S and supplied the Inbicon pretreated material. The rest of the authors declare they have no competing interests regarding this paper.

## Authors’ contributions

MATH provided experimental coordination, performed lab-scale pretreatments, dewaxing, enzymatic hydrolyses and chemical composition analyses, including HPLC, and performed LM, SEM and AFM imaging and contributed in collecting CLSM images. BJH incubated the enzymes with fluorescent markers, performed CLSM imaging and participated in continual manuscript revisions. KKM and MDJ performed enzymatic hydrolyses and following HPLC analysis and participated in manuscript revisions. BJ performed LM imaging, checked plant anatomy concepts and made manuscript revisions. KSJ delivered the enzymes used for CLSM analysis, identified the fluorescent markers used in the experiments and provided manuscript revisions. LGT provided IR-analyses, experimental coordination and design and substantial manuscript revisions throughout the whole process. All authors have read and approved the final manuscript.
